# Lysine Demethylase 1 Has Demethylase-Dependent and Non-Canonical Functions in Myofibroblast Activation in Systemic Sclerosis

**DOI:** 10.3390/cells14060433

**Published:** 2025-03-14

**Authors:** Christopher W. Wasson, Esther Perez Barreiro, Francesco Del Galdo, Natalia A. Riobo-Del Galdo

**Affiliations:** 1 Leeds Institute of Rheumatic and Musculoskeletal Medicine, Faculty of Medicine and Health, University of Leeds, Leeds LS2 9JT, UK; c.w.wasson@leeds.ac.uk (C.W.W.); f.delgaldo@leeds.ac.uk (F.D.G.); 2 School of Molecular and Cellular Biology, Faculty of Biological Sciences, University of Leeds, Leeds LS2 9JT, UK; bsepb@leeds.ac.uk; 3 Scleroderma Programme, NIHR Leeds Musculoskeletal Biomedical Research Centre, Leeds LS7 4SA, UK; 4 Leeds Institute of Medical Research, Faculty of Medicine and Health, University of Leeds, Leeds LS2 9JT, UK; 5 Astbury Centre for Structural Molecular Biology, University of Leeds, Leeds LS2 9JT, UK

**Keywords:** systemic sclerosis, fibrosis, LSD1, HOTAIR

## Abstract

Systemic sclerosis (SSc) is an autoimmune disease of unknown aetiology characterised by vasculopathy with progressive fibrosis of the skin and internal organs. Tissue fibrosis is driven by activated fibroblasts (myofibroblasts) with exacerbated contractile and secretory properties. We previously reported that the long non-coding RNA HOTAIR is a key driver of SSc fibroblast activation. HOTAIR interacts with the chromatin modifiers, the polycomb repressor complex (PRC2) and coREST complex, promoting expression of pro-fibrotic genes. In this study, we show that acute activation of dermal fibroblasts from healthy subjects or SSc patients with transforming growth factor-β and other fibrotic stimuli requires the activity of the lysine-specific demethylase 1 (LSD1) subunit of the co-REST complex. Unexpectedly, LSD1 catalytic activity plays a minor role in fibrotic gene expression in HOTAIR-overexpressing fibroblasts and in maintenance of the stable myofibroblast phenotype of SSc fibroblasts. However, silencing of LSD1 in SSc fibroblasts has a profound effect on pro-fibrotic gene expression, supporting a non-canonical scaffolding function. Our study shows for the first time an essential non-canonical role for LSD1 in pro-fibrotic gene expression in SSc; however, given that this function is insensitive to LSD1 inhibitors, the therapeutic opportunities will depend on future identification of a targetable mediator.

## 1. Introduction

Systemic sclerosis (SSc) is an autoimmune disease characterised by diffuse vasculopathy and progressive fibrosis of the skin and internal organs [1]. SSc has the highest mortality among rheumatic diseases [1]. Once established, fibrosis is deemed irreversible, causing organ dysfunction. Therefore, identification of therapeutic targets that reduce development of fibrosis or promote resolution of fibrosis is desirable.

Tissue fibrosis is mediated by a mesenchymal cell phenotype with high contractility and secretory capacity, the myofibroblast, which deposits large amounts of collagen in the extracellular matrix and increases tissue stiffness [2]. The myofibroblast phenotype can be acquired by activation of tissue-resident fibroblasts with transforming growth factor-β (TGF-β) or activation of other pro-fibrotic signalling pathways, such as Wnt, Notch, and Hedgehog [3,4,5,6]. Other cell types can transdifferentiate into myofibroblasts, including epithelial and endothelial cells through processes known as epithelial-to-mesenchymal transition (EMT) and endothelial-to-mesenchymal transition (EndoMT) [4]. The processes of EMT and EndoMT first require repression of E-cadherin and VE-cadherin by upregulation of EMT transcription factors such as Snail, Slug, Zeb1, and Twist, followed by upregulation of mesenchymal markers [7]. Repressive epigenetic marks that decommission promoters play central roles in orchestrating the differentiation and maintenance of myofibroblasts.

We have previously reported that the long non-coding RNA HOTAIR is aberrantly upregulated in SSc skin and isolated dermal fibroblasts and that silencing of HOTAIR significantly reduces profibrotic marker expression in SSc fibroblasts [8]. HOTAIR binds to the polycomb repressor complex (PRC2) to guide H3K27me3 methylation to specific promoters, resulting in their transcriptional repression [9]. However, genomic redistribution of PRC2 by HOTAIR scaffolding leads to reduced PRC2 association with some promoters, leading to upregulation of genes involved in embryonic fibroblast identity, EMT, migration, and invasion [10]. In addition, HOTAIR/PRC2 could repress expression of transcriptional repressors that regulate those processes. Indeed, we reported that forced expression of HOTAIR in dermal fibroblasts caused an upregulation of pro-fibrotic markers leading to fibroblast activation through its ability to regulate the PRC2 complex [8]. Inhibition of EZH2, the enzymatic subunit of PRC2, blocks SSc fibroblast activation [8,11,12]. Furthermore, our studies showed that HOTAIR positively regulates a number of pro-fibrotic signalling pathways in SSc fibroblasts, including the Notch, Wnt, and Hedgehog signalling pathways, leading to upregulation of the transcription factor GLI2, which is necessary for fibrosis [8,11].

In addition to its well-characterised role in regulating the PRC2 complex, HOTAIR can bind to Lysine Demethylase 1 through its 3′ domain (LSD1) [9,13], an enzyme that removes monomethyl and dimethyl groups from H3K4, decommissioning enhancers and promoters [14]. HOTAIR-bound LSD1 is part of the transcriptional repressor complex co-REST/REST, but not with other binding partners of LSD1 [9]. There is evidence to suggest HOTAIR can recruit both PRC2 and the co-REST complexes to the same promoters [9]. That study demonstrated that of all promoters co-occupied by SUZ12 (a subunit of the PRC2) and LSD1, about 40% showed simultaneous loss of PRC2 and LSD1, while 33% showed loss of LSD1 and 16% exhibited loss of SUZ12 upon knockdown of HOTAIR in foreskin fibroblasts [9]. The importance of the LSD1/co-REST complex, which also contains HDAC1/2 [15], has been shown in some models of EMT in the context of fibrosis [16] and in HOTAIR-associated cell migration [17].

The aim of this study was to determine if LSD1 plays a role in SSc fibroblast activation and investigate its dependency of HOTAIR. For this, we first investigated the role of LSD1 demethylase activity in healthy dermal fibroblast activation, using an irreversible inhibitor, and the combined activity-dependent and non-canonical roles of LSD1, using siRNA to silence LSD1 expression. Next, we investigated if the function of LSD1 is conserved in SSc myofibroblasts and if its role is dependent on HOTAIR. Interestingly, LSD1 functions in healthy and SSc fibroblasts seem to be mostly independent from HOTAIR.

## 2. Materials and Methods

### 2.1. Patient Cell Lines

Full-thickness skin biopsies were surgically obtained from the forearms of four adult patients with recent onset SSc, defined as a disease duration of less than 18 months from the appearance of clinically detectable skin induration. All patients satisfied the 2013 ACR/EULAR criteria for the classification of SSc and had diffuse cutaneous clinical subset as defined by LeRoy et al. [18]. The patients had an mRSS score ranging from 2 to 22; three of four were Scl70-positive and had concurrent mild ling fibrosis, while one patient had severe lung fibrosis. Two healthy controls were included in the study. All participants provided written informed consent to participate in the study. Informed consent procedures were approved by NRES-011NE to FDG. Fibroblasts were isolated and established as previously described [19]. Primary cells were immortalised using human telomerase reverse transcriptase (hTERT) to produce healthy control hTERT and SSc hTERT.

### 2.2. Lentiviral Transduction

Fibroblasts (FBs) were grown from healthy control forearm biopsies and immortalised using retrovirus-expressing human telomerase (hTERT) as previously outlined [19]. HOTAIR expression was then induced by transduction with GIPZ lentiviruses carrying HOTAIR gene sequence or scrambled RNA sequence as control in frame with the puromycin resistance gene and the GFP fluorochrome gene (Open Biosystems, Surrey, UK). For this purpose, FBs were seeded at 50% confluence and infected with lentiviral particles in serum-free DMEM and incubated for 6 h, after which an additional 1 mL of DMEM containing 10% FCS was added, and the cells were incubated for a further 72 h. Stably transduced FBs were positively sorted for GFP fluorescence employing Fluorescence-Activated Cell Sorting in sterile conditions (BD INFLUX, Warsaw, Poland). Positively sorted cells were further selected in media containing 1.0 μg/mL puromycin (Life Technologies, Paisley, UK) for 10 days.

### 2.3. Small-Molecule Inhibitors

GSK-126 is an EZH2 methylation transferase inhibitor. It was used at a final concentration of 5 μM and purchased from LKT Laboratories, St. Pauls, MN, USA (G7340). GSK-LSD1 is an LSD1 inhibitor that was purchased from Sigma Aldrich, Sofia, Bulgaria (SML1072). It was used at a final concentration of 10 μM. All inhibitors were added to fibroblasts in complete media for 48 h at 37 °C in a 5%CO_2_ atmosphere.

### 2.4. Treatment of Fibroblasts with Pro-Fibrotic Factors

Healthy dermal fibroblasts were serum-starved for 24 h in Dulbecco Modified eagle media (DMEM) containing 0.5% FBS and stimulated with either 10 ng/mL TGF-β1 (R&D systems, Minneapolis, MN, USA), 100 ng/mL Wnt-3a (R&D systems), 100 nM SMO agonist (SAG), 50 ng/mL IL-6, or 10 μM LPA for 48 h.

### 2.5. siRNA Transfections

A pool of four siRNAs specific for different regions of LSD1 or a negative control scrambled siRNA (Qiagen) were transfected into fibroblasts using Lipofectamine 2000 (Thermo Fisher, Waltham, MO, USA). Fibroblasts were incubated for 48 h prior to harvesting.

### 2.6. Western Blotting

Total proteins were extracted from fibroblasts in RIPA buffer and resolved by SDS-PAGE (10–15% Tris-Glycine). Proteins were transferred onto Hybond nitrocellulose membranes (Amersham biosciences, Chandler, AZ, USA) and probed with antibodies specific for collagen type I (Santa Cruz, Dallas, TX, USA), alpha smooth muscle actin (Abcam, Cambridge, UK), NOTCH1 (Cell signalling, Danvers, CO, USA), β-catenin (Cell signalling), CTGF (Abcam), SMAD3 (Cell signalling), pSMAD3 (Cell signalling), GLI2 (R&D systems), and β-actin (Sigma Aldrich). Immunoblots were visualised with species-specific HRP-conjugated secondary antibodies (Sigma Aldrich) and ECL (Thermo/Pierce, Waltham, MO, USA) on a BioRad ChemiDoc imaging system.

### 2.7. Quantitative Real-Time PCR

RNA was extracted from cells using commercial RNA extraction kits (Zymo Research, Irvine, CA, USA). RNA (1 ug) was reverse-transcribed using cDNA synthesis kits (Thermo-Fisher). QRT-PCRs were performed using SyBr Green PCR kits on a Thermocycler with primers specific for *ACTA2* (forward: TGTATGTGGCTATCCAGGCG; reverse: AGAGTCCAGCACGATGCCAG), *COL1A2* (forward: GATGTTGAACTTGTTGCTGAGC; reverse: TCTTTCCCCATTCATTTGTCTT), *NOTCH1* (forward: CCAGAACTGTGAGGAAAATATCG; reverse: TCTTGCAGTTGTTTCCTGGAC), *HES1* (forward: TACCCAGCCAGTGTCAAC; reverse: CAGATGCTGTCTTTGGTTTATCC), and *GAPDH* (forward: ACCCACTCCTCCACCTTTGA; reverse: CTGTTGCTGTAGCCAAATTCGT). Data were analysed using the ΔΔCt method using *GAPDH* a housekeeping gene.

### 2.8. Immunofluorescence

Fibroblasts were seeded onto coverslips. After stimulation/treatment, the cells were fixed in 4% paraformaldehyde and permeabilised with 0.1% trition-x20 for 10 min. The cells were stained with an alpha-SMA or pSMAD3 antibody and visualised with a secondary antibody conjugated to alexa-594. Nuclei were visualised by DAPI contained within the mounting media. Scale bars represent 20 μm.

### 2.9. Statistical Analysis

Data are presented as the mean ± standard error. Statistical analysis was performed using a two-tailed, unpaired Student’s *t*-test for comparisons of two groups. Multiple groups were analysed by one-way ANOVA, followed by post hoc multiple comparisons using GraphPad Prism 10.4.1.

## 3. Results

### 3.1. LSD1 Activity Is Required for Maximal TGF-β-Induced Dermal Fibroblast Activation

**Figure 1:**
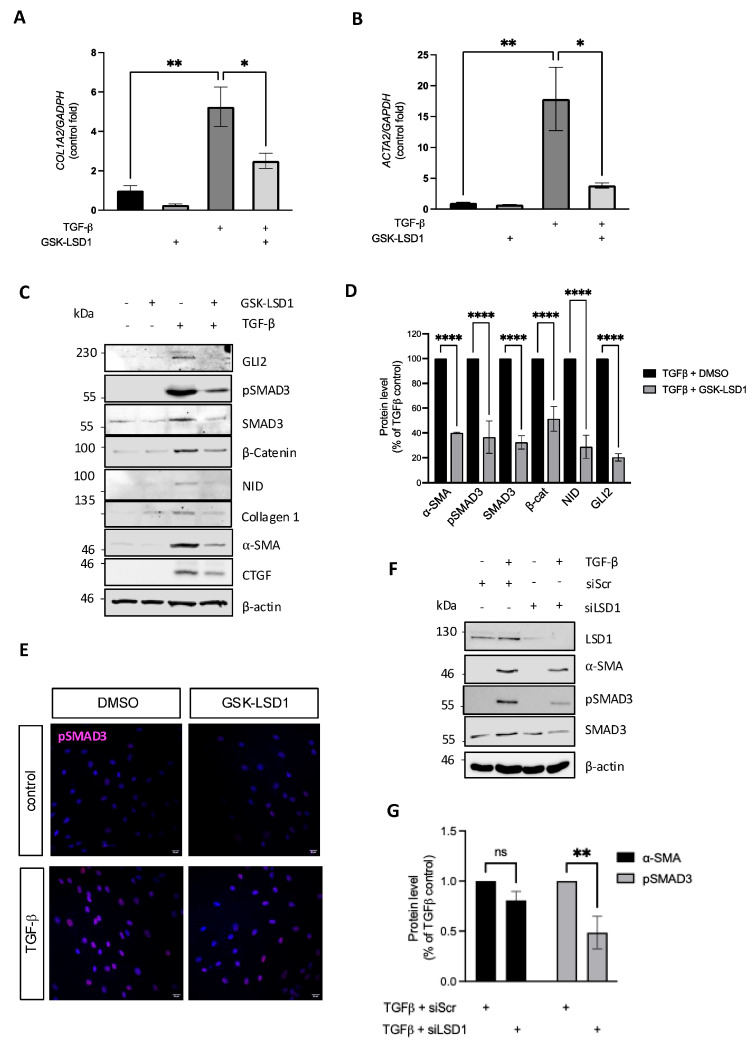
LSD1 activity is required for maximal TGF-β-induced dermal fibroblast activation. Healthy dermal fibroblasts were stimulated with 10 ng/mL TGF-β for 48 h or left untreated. In addition, the fibroblasts were treated with the LSD1 inhibitor GSK-LSD1 (10 µM) or DMSO as control. *COL1A2* (**A**) and *ACTA2* (**B**) transcript levels were assessed by qPCR. The graphs represent the mean and standard error for three independent experiments. (**C**) Protein levels of collagen type I, α-SMA, GLI2, CTGF, β-catenin, NID, pSMAD3, and total SMAD3 were assessed by Western blot, using β-actin as loading control. A representative experiment is shown. (**D**) Densitometric analysis of all fibrotic markers in TGF-β-stimulated samples with or without GSK-LSD1 normalised to β-actin levels. The data represent 3 independent experiments, with the intensity of the bands in the TGF-β-only sample set as 100%. (**E**) pSMAD3 staining (red) of healthy dermal fibroblasts stimulated with 10 ng/mL TGF-β in the presence or absence of 10 µM GSK-LSD1 for 48 h. Nuclei are counterstained with DAPI. (**F**) Protein was extracted from healthy dermal fibroblasts transfected with siRNA specific to LSD1 (siLDS1) or a scrambled negative control (siSrc) after stimulation with 10 ng/mL TGF-β for 48 h. The protein levels of α-SMA, pSMAD3, total SMAD3, and LSD1 were assessed by Western blot, using β-actin as a loading control. (**G**) Densitometric analysis of α-SMA and pSMAD3 levels normalised to β-actin. The data represent 3 independent experiments, with the intensity of the bands in the TGF-β-treated siScr cells set as 100%. All graphs represent the mean +/− standard error, while ns *p* > 0.05; * *p* < 0.05; ** *p* < 0.01; **** *p* < 0.0001 in one-way ANOVA followed by multiple comparisons (*t*-tests).

Following up on reports of a requirement of LSD1 activity for EMT and activation of renal fibroblasts with TGF-β [20], we investigated its role in normal human dermal fibroblasts. Cells were stimulated with TGF-β in the presence and absence of the mechanism-based irreversible LSD1 inhibitor GSK-LSD1 [21]. Interestingly inhibition of LSD1 blocked TGF-β induction of *COL1A2* and *ACTA2* (encoding α-smooth muscle actin) transcription (Figure 1A,B), as well as collagen type I, connective tissue growth factor (CTGF), and α-SMA protein expression (Figure 1C,D). TGF-β signalling is mediated by phosphorylation and activation of the SMAD2/3 transcription factors. As expected, phospho-SMAD3 (pSMAD3) levels were increased in the TGF-β-stimulated fibroblasts and localised to the nucleus (Figure 1C,E), and its activation was reduced by the LSD1 inhibitor. Inhibition of LSD1 in TGF-β-stimulated fibroblasts also reduced the levels of total SMAD3, which were upregulated after 24 h stimulation with TGF-β. This suggests LSD1 might be important for regulating SMAD3 expression, leading to secondary changes in pSMAD3 levels when dermal fibroblasts are stimulated with TGF-β. The reduction in total SMAD3 levels when LSD1 was inhibited is exclusive to TGF-β-stimulated fibroblasts, as total SMAD3 was unaffected in unstimulated fibroblasts treated with the LSD1 inhibitor (Figure 1C). The efficient attenuation of SMAD3 activation by the LSD1 inhibitor reduced the TGF-β-induced upregulation of GLI2 and β-catenin and reduced formation of the Notch intracellular domain (NID) (Figure 1C,D). Upregulation of those transcriptional regulators is key for promoting the expression of pro-fibrotic genes, as we have previously shown [8]. Finally, we validated the results obtained using the LSD1 inhibitor by silencing LSD1 expression in normal dermal fibroblasts with siRNA. Cells transfected with siLSD1 had a 90% reduction in LSD1 at the protein level compared to cells transfected with a scrambled control RNA (siScr) (Figure 1F). As expected, silencing of LSD1 significantly attenuated the response to TGF-β, as evidenced by significantly reduced SMAD3 phosphorylation and a trend to reduction in α-SMA expression (Figure 1F,G). The reduction in pSMAD3 observed in the fibroblasts stimulated with TGF-β plus the LSD1 siRNA is likely due to a reduction in the total levels of SMAD3 in these cells. This is similar to the effect seen with the LSD1 inhibitor (Figure 1C). Altogether, these results indicate that canonical TGF-β signalling and the fibrotic response in dermal human fibroblasts require LSD1 demethylase activity.

### 3.2. Inhibition of LSD1 Demethylase Activity Attenuates Fibroblast Activation in Response to Multiple Fibrotic Stimuli

**Figure 2:**
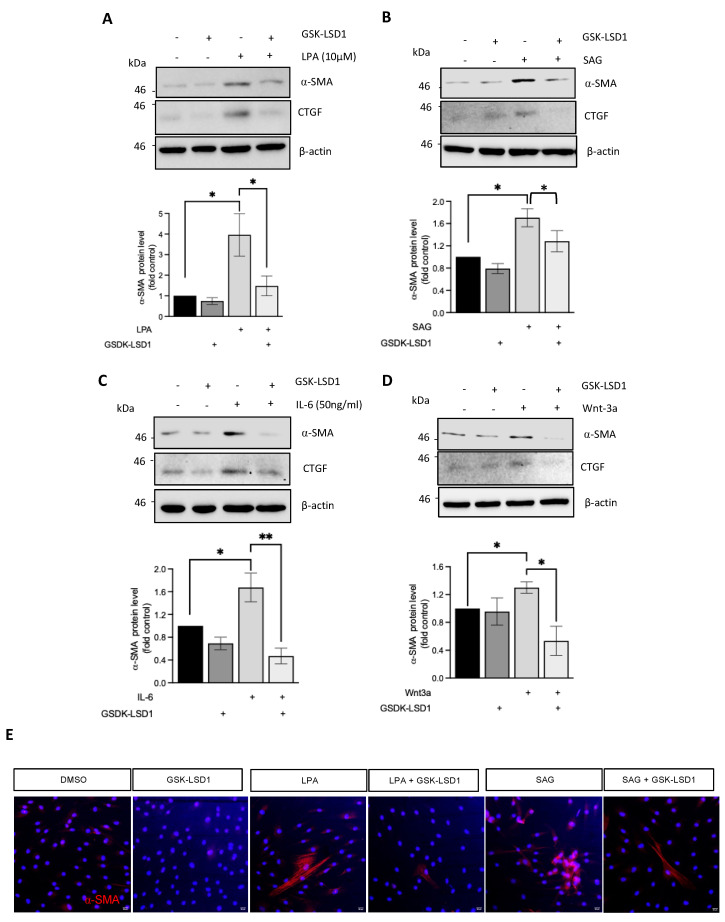
LSD1 catalytic activity plays a role in fibroblast activation induced through fibrotic stimuli. Induction of α-SMA at the protein level from healthy dermal fibroblasts stimulated with different pro-fibrotic stimuli for 48 h in the absence or presence of GSK-LSD1 was determined by Western blot. Data from a densitometric analysis of α-SMA levels normalised to β-actin from 3 independent experiments are shown as mean +/− SEM. * *p* < 0.05; ** *p* < 0.01. (**A**) Effect of GSK-LSD1 on α-SMA and CTGF induction by LPA. (**B**) Effect of GSK-LSD1 on α-SMA and CTGF induction with SAG. (**C**) Effect of GSK-LSD1 on α-SMA and CTGF induction with IL-6. (**D**) Effect of GSK-LSD1 on α-SMA and CTGF induction with Wnt3a. (**E**) α-SMA staining of healthy dermal fibroblasts (red) stimulated with LPA or SAG +/− GSK-LSD1. Nuclei were counterstained with DAPI.

The data above demonstrate that LSD1 regulates pro-fibrotic gene expression upon activation by TGF-β. While TGF- β is the chief pro-fibrotic growth factor, we and others have previously reported important contributions of the canonical Hedgehog and Wnt pathways to fibroblast activation, as well as a pro-fibrotic response to interleukin-6 (IL-6) and lysophosphatidic acid (LPA). We thus investigated the role of LSD1 in normal dermal fibroblasts stimulated with Wnt3a, the Hedgehog pathway agonist SAG, IL-6, and LPA in the presence or absence of GSK-LSD1. Inhibition of LSD1 activity blocked the induction of α-SMA and CTGF in response to LPA (Figure 2A,E), SAG (Figure 2B,E), IL-6 (Figure 2C), and Wnt3a (Figure 2D,E) in healthy fibroblasts. These data suggest that LSD1 demethylase activity and, likely, the co-REST complex have a role in fibroblast activation in response to a range of fibrotic stimuli.

### 3.3. SSc Dermal Fibroblasts Require LSD1 Activity for Maximal Response to TGF-β but Not for Maintenance of the Myofibroblastic Phenotype

**Figure 3:**
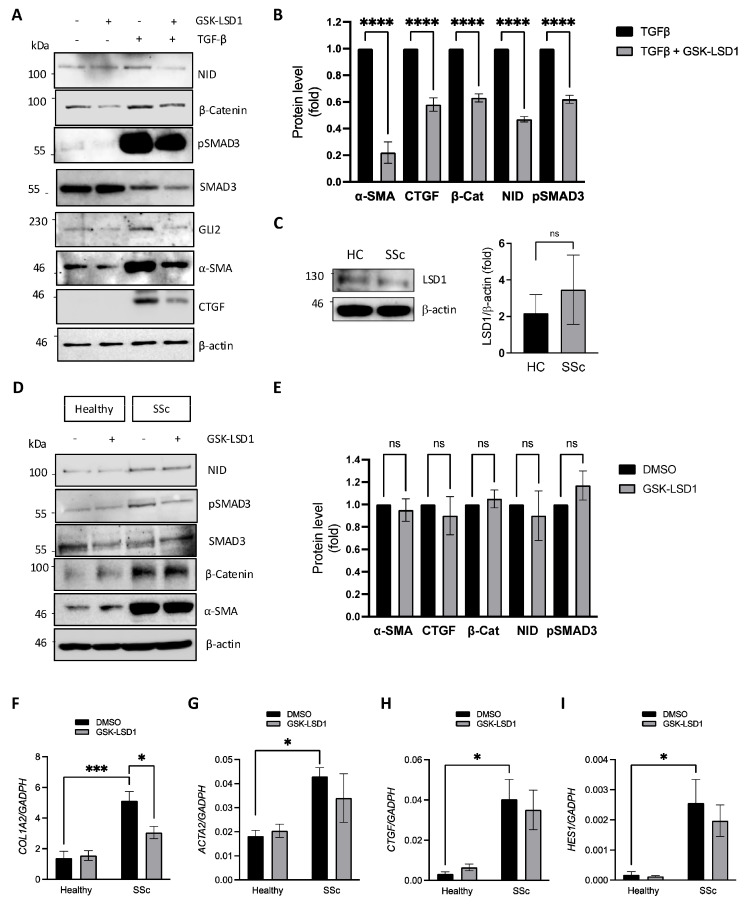
LSD1 activity is dispensable for basal fibrotic marker expression but is required for TGF-β signalling in SSc fibroblasts. (**A**) SSc dermal fibroblasts were stimulated or not stimulated with 10 ng/mL TGF-β in the presence or absence of 10 µM GSK-LSD1 for 48 h. NID, β-Catenin, pSMAD3, total SMAD3, GLI2, α-SMA, and CTGF protein levels were assessed by Western blot, using β-actin as loading control. (**B**) Densitometric analysis of pSMAD3, α-SMA, CTGF, NID, and β-catenin protein level changes in SSc fibroblasts treated with TGF-β in the presence or absence of GSK-LSD1 (n = 3). Data are derived from 3 independent experiments. (**C**) LSD1 protein levels were assessed in healthy (n = 3) and SSc (n = 3) dermal fibroblast protein lysates. (**D**) Healthy and SSc dermal fibroblasts were treated with DMSO or 10 µM GSK-LSD1 for 48 h, followed by analysis of protein levels of NID, β-catenin, pSMAD3, total SMAD3, and α-SMA by Western blot, using β-actin as loading control. (**E**) Densitometry analysis of α-SMA, CTGF, β-catenin, NID, and pSMAD3 protein levels in SSc fibroblasts treated with GSK-LSD1 (n = 3). Data are derived from 3 independent experiments. In addition, RNA was extracted from healthy and SSc fibroblasts treated in the same manner followed by relative quantification of *COL1A2* (**F**), *ACTA2* (**G**), *CTGF* (**H**), and *HES1* (**I**) transcript levels via qPCR analysis. All graphs represent the mean +/− standard error for three independent experiments. ns, not significant, * *p* < 0.05, *** *p* < 0.001, **** *p* < 0.0001 in one-way ANOVA followed by multiple comparisons (*t*-tests).

Given the important role of LSD1 activity in regulating pro-fibrotic signalling in healthy dermal fibroblasts, we sought to determine the role of LSD1 in SSc patient-derived fibroblasts, which are primed to respond to TGF-β stimulation. Treatment of SSc fibroblasts with TGF-β resulted in very high SMAD3 phosphorylation and subsequently increased NID, β-catenin, GLI2, α–SMA, and CTGF levels (Figure 3A). Activation of SMAD3 and upregulation of α–SMA, CTGF, β-catenin, and NID were significantly reduced by GSK-LSD1 (Figure 3A,B). Inhibition of LSD1 in TGF-β-stimulated fibroblasts reduced the levels of total SMAD3, suggesting LSD1 is important for regulating SMAD3 expression, leading to changes in pSMAD3 levels when SSc dermal fibroblasts are stimulated with TGF-β. This finding is similar to the results described in healthy dermal fibroblasts (Figure 1C) and suggests that LSD1 has an essential role in maximal TGF-β/SMAD3 signalling in SSc and healthy dermal fibroblasts.

Because SSc fibroblasts have a heightened basal expression of fibrotic markers even in the absence of exogenous TGF-β, we also studied the role of LSD1 on pro-fibrotic gene expression in basal conditions. First, we assessed the expression profile of LSD1 in healthy and SSc dermal fibroblasts. LSD1 levels were similar between the healthy and SSc dermal fibroblasts (Figure 3C). Unexpectedly, inhibition of the LSD1 demethylase activity with GSK-LSD1 in SSc fibroblasts did not alter NID production, pSMAD3, α-SMA, or β-catenin at the protein level (Figure 3D,E). In agreement, LSD1 inhibition in SSc cells had no effect on *ACTA2*, *CTGF,* or *HES1* transcript levels (Figure 3G–I), although it significantly reduced *COL1A2* expression (Figure 3F). These results indicate that LSD1 demethylase activity is required for maximal response to TGF-β both in normal and SSc fibroblasts but that its activity is dispensable for stable basal overexpression of pro-fibrotic markers and mediators in SSc.

### 3.4. LSD1 Supports the SSc Myofibroblast Phenotype Through Non-Canonical Functions

**Figure 4:**
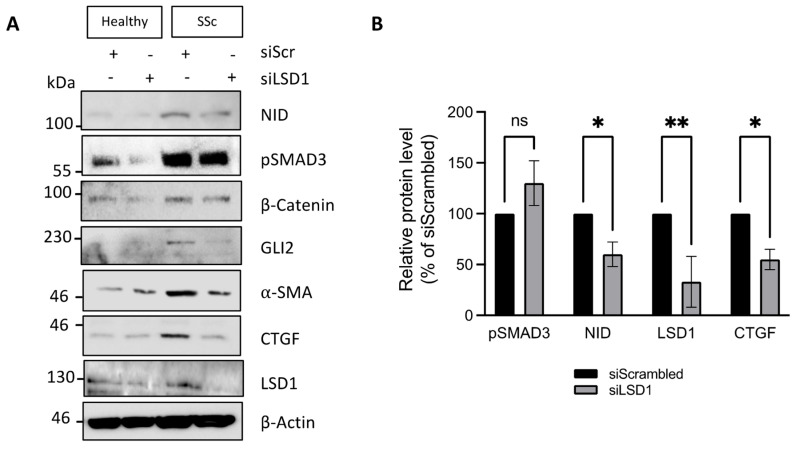
LSD1 functions in a demethylase-independent manner to support maintenance of the fibrotic phenotype in SSc fibroblasts. (**A**) Protein was extracted from healthy and SSc dermal fibroblasts transfected with siRNA specific for LSD1 (siLSD1) or a scrambled negative control (siSrc) for 48 h. NID, pSMAD3, β-catenin, GLI2, α-SMA, CTGF, and LSD1 levels were assessed by Western blot, using β-actin as loading control. (**B**) Densitometric quantification of pSMAD3, NID, LSD1, and CTGF levels normalised to β-actin in SSc fibroblasts transfected with control siScr or siLSD1. Protein band intensity in the control siSrc condition was set to 100%. The graph represents the mean +/− standard error of 3 independent experiments. ns *p* > 0.05; * *p* < 0.05; and ** *p* < 0.01 in *t*-tests.

The experiments with GSK-LSD1 in SSc fibroblasts suggest that LSD1 has a limited role in regulating pro-fibrotic gene expression in SSc dermal fibroblasts. An alternative interpretation is that LSD1 demethylase activity, as part of the co-REST complex, is not required for maintenance of the fibrotic phenotype. To test the possibility of a non-enzymatic role of LSD1 in SSc, we assessed pro-fibrotic gene expression in healthy and SSc dermal fibroblasts transfected with LSD1 siRNA or a scrambled siRNA control. The LSD1 siRNA treatment reduced LSD1 protein levels by 70% in both healthy and SSc fibroblasts (Figure 4A,B). Interestingly, unlike the irreversible LSD1 inhibitor, silencing of LSD1 reduced expression of NID and CTGF (Figure 4A,B). In addition, LSD1 siRNA reduced β-catenin, α-SMA, and GLI2 expression levels (Figure 4A). The lack of effect of an irreversible LSD1 inhibitor on basal fibrotic gene expression but for *COL1A2* in SSc fibroblasts, together with the more profound changes caused by knockdown of LSD1, suggests a non-canonical, demethylase-independent role for LSD1 in maintenance of pro-fibrotic gene expression in SSc dermal fibroblasts.

### 3.5. Inhibition of LSD1 Activity Attenuates Pro-Fibrotic Gene Expression in HOTAIR Expressing Fibroblasts

**Figure 5:**
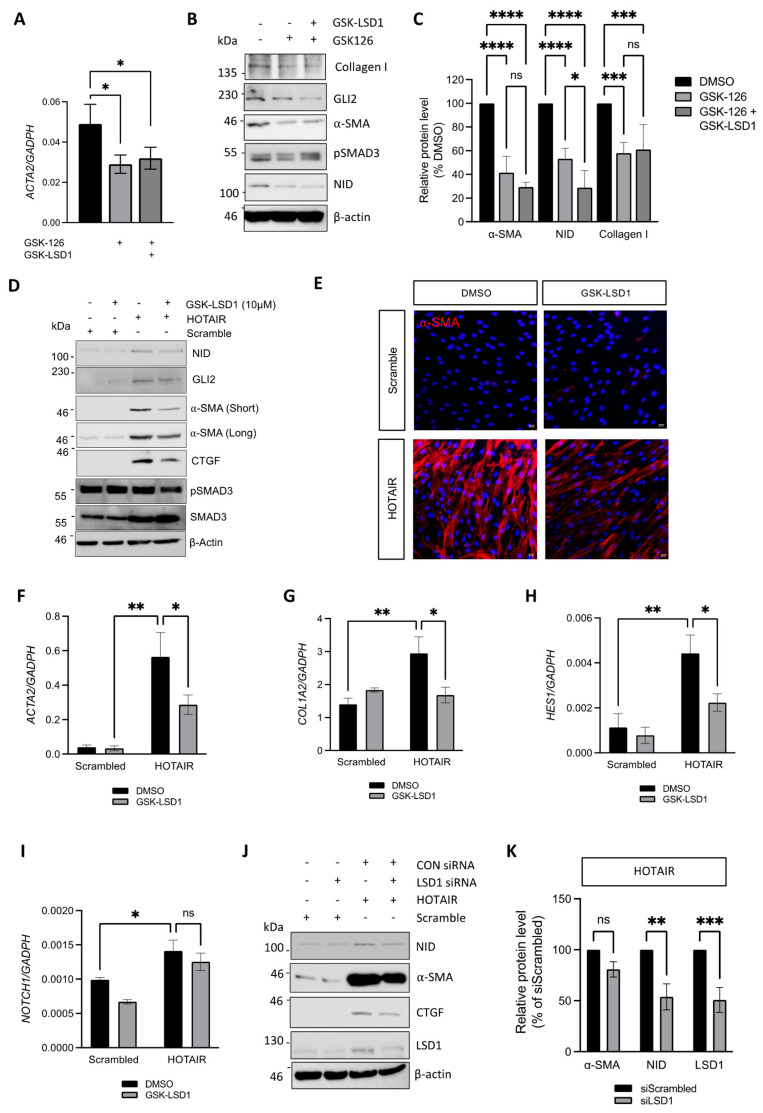
LSD1 demethylase activity contributes to HOTAIR-induced fibroblast activation. SSc dermal fibroblasts were treated with DMSO, GSK126 alone, or GSK-126 plus GSK-LSD1 for 48 h, followed by determination of *ACTA2* transcript level by qPCR. (**A**) Protein levels of collagen type I, GLI2, α-SMA, pSMAD3, and NID, assessed by Western blot using β-actin as loading control (**B**). (**C**) Densitometric analysis of changes in α-SMA, NID, and collagen I expression in SSc fibroblasts by the EZH2 and LSD1 inhibitors compared to vehicle control. Protein quantification was normalised to β-actin. (**D**) Dermal fibroblasts transduced with a lentiviral vector encoding HOTAIR or a scrambled RNA (Scramble) were treated with DMSO or GSK-LSD1 for 48 h, followed by lysis and protein determination of NID, GLI2, α-SMA, CTGF, pSMAD3, and total SMAD3 by Western blot, using β-actin as loading control. (**E**) HOTAIR or Scramble fibroblasts treated (or not) with GSK-LSD1 were stained for assessment of α-SMA levels (red) and counterstained with DAPI. The transcript levels of fibrotic markers and mediators in HOTAIR or Scramble fibroblasts treated (or not) with GSK-LSD1 were assessed by qPCR: *ACTA2* (**F**)*, COL1A2* (**G**), *HES1* (**H**), and *NOTCH1* (**I**). (**J**) HOTAIR or Scramble fibroblasts were transfected with control siScr or siLSD1, followed by determination of protein levels of NID, α-SMA, CTGF, pSMAD3, and LSD1 by Western blot, using β-actin as loading control. (**K**) Densitometric quantification of protein levels of α-SMA, NID, and LSD1 in HOTAIR-expressing fibroblasts transfected with control siScr or siLSD1. All graphs represent the mean +/− standard error for 3 independent experiments. ns *p* > 0.05, * *p* < 0.05, ** *p* < 0.01, *** *p* < 0.001 and **** *p* < 0.0001 in one-way ANOVA followed by multiple comparisons (*t*-tests).

The previous finding was unexpected given the upregulation of HOTAIR in SSc fibroblasts, which instructs LSD1/co-REST and EZH2/PRC2 localisation to specific genomic loci to increase repressive (demethylated) H3K4 and H3K27me3 epigenetic marks. Therefore, we speculated that LSD1 activity could be relevant for pro-fibrotic gene expression only in conjunction with EZH2 inhibition. To test this possibility, we treated SSc fibroblasts with the EZH2 inhibitor GSK-126 alone or in combination with GSK-LSD1. Interestingly, while GSK-126 reduced *ACTA2* mRNA expression (Figure 5A) and protein levels of collagen type I, GLI2, NID, and α-SMA (Figure 5A–C), as we previously reported, inhibition of LSD1 activity did not further reduce any fibrotic marker, with the exception of NID (Figure 5A–C). This finding is in agreement with the observed lack of effect of LSD1 inhibition in the SSc fibrotic phenotype (described above).

However, we have previously reported that overexpression of HOTAIR in healthy dermal fibroblasts increases fibrotic marker expression, in particular that of α-SMA [8]. Therefore, we sought to investigate whether LSD1 activity has a different role in HOTAIR-overexpressing fibroblasts than in SSc fibroblasts. Scramble control or HOTAIR-expressing fibroblasts were treated with GSK-LSD1, an inhibitor of LSD1 demethylase activity, for 48 h. As previously described, HOTAIR-overexpressing fibroblasts had increased expression of α-SMA, CTGF, and GLI2, as well as increased levels of NID, compared to the scrambled control cells (Figure 5D). Treatment of HOTAIR fibroblasts with GSK-LSD1 partially reduced α-SMA, CTGF, and GLI2 at the protein level (Figure 5D,E). In agreement, the LSD1 inhibitor reduced mRNA levels of *ACTA2*, *COL1A2*, and the Notch-responsive gene *HES1* at the transcript level (Figure 5F–H), without altering *NOTCH1* transcript levels (Figure 5I).

We observed similar results by silencing LSD1 expression with siRNA in HOTAIR-expressing fibroblasts. LSD1 siRNA reduced LSD1 protein levels by ~50% in both the scramble control and HOTAIR-expressing fibroblasts (Figure 5J,K). The ability of HOTAIR to induce α-SMA expression and activate Notch signalling was reduced by ~20% and ~50%, respectively, despite the incomplete silencing of LSD1 (Figure 5J,K). Altogether, these results demonstrate that, in addition to the previously reported role of EZH2 and PCR2, LSD1 activity is required for the fibrotic phenotype of HOTAIR-overexpressing fibroblasts. However, SSc fibroblasts exhibit an additional requirement of LSD1 protein that is independent of HOTAIR and seems to be unrelated to its demethylase activity.

## 4. Discussion

**Figure 6:**
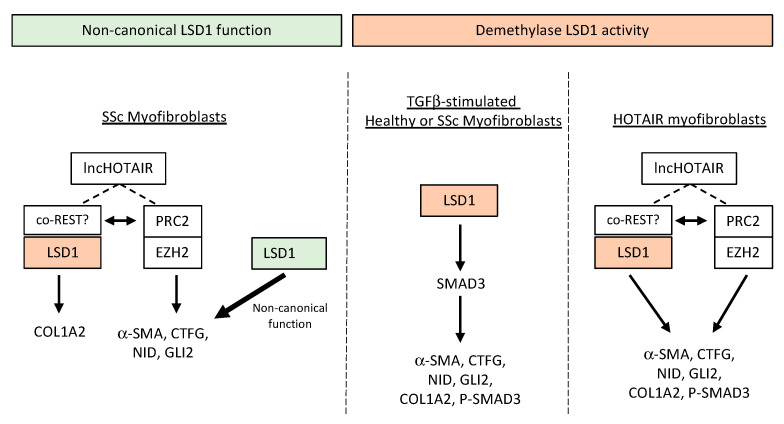
The role of the LSD1 in activated myofibroblasts of different origin. (**Right**) Schematic model of the function of enzymatic LSD1 in TGF-β-stimulated normal or SSc fibroblasts and in HOTAIR-expressing fibroblasts, presumably through complex formation with co-REST (orange). (**Left**) Non-enzymatic activity of LSD1 regulating pro-fibrotic gene expression is more important in maintenance of the primed SSc fibrotic phenotype (green).

In this study, we uncovered an important role of LSD1 in dermal fibroblast activation by multiple fibrotic stimuli and in maintenance of the fibrotic phenotype in myofibroblasts isolated from SSc patients. Remarkably, the role of LSD1 in each context is facilitated by different mechanisms (Figure 6). Firstly, we have shown that LSD1 enzymatic activity is essential for induction of pro-fibrotic gene markers and mediators in response to TGF-β. Importantly, this role of LSD1 is conserved in SSc myofibroblasts, which express higher levels of fibrotic markers and mediators but are also primed to respond to TGF-β. Previous studies have shown LSD1 has important roles in regulating renal, cardiac, and pulmonary fibrosis [22,23,24], suggesting a ubiquitous function of LSD1. Interestingly inhibition of LSD1 in healthy fibroblasts stimulated with Wnt-3a, SAG, IL-6, or LPA resulted in a reduction in the expression of the pro-fibrotic markers analysed. This suggests that the LSD1/co-REST complex drives pro-fibrotic gene expression in response to fibrotic stimuli.

Remarkably, we found that LSD1 demethylase activity is dispensable for maintenance of the pro-fibrotic gene expression in SSc fibroblasts, which was unexpected for two reasons: the heightened TGF-β signalling and the upregulation of the lncRNA HOTAIR, which localises chromatin-repressive modifier enzymes, including LSD1/coREST, to specific regions of the genome. The use of a mechanism-based irreversible LSD1 inhibitor, GSK-LSD1, which increased H3K4me2 levels and efficiently blocked TGF-β signalling, rules out an enzymatic role of LSD1 in basal SSc myofibroblast maintenance. Instead, silencing of LSD1 expression, even partially, was sufficient to reduce expression of fibrotic markers and mediators in SSc fibroblasts in basal conditions. This supports a different mechanism of action of LSD1 in SSc independent of its demethylase activity, which may or may not require regulation of the coREST complex. Further support for this conclusion was obtained by the lack of additive or synergistic inhibition of pro-fibrotic markers in SSc fibroblasts treated with an EZH2/PRC2 inhibitor and an LSD1 inhibitor.

Demethylase-independent roles of LSD1 have been reported in the literature. A recent study of embryonic stem cell differentiation compared LSD1 knockout cells with cells expressing a catalytically inactive mutant of LSD1 and observed a much more profound upregulation of global enhancers by loss of LSD1 protein expression than by loss of its demethylase activity [25]. This study further ruled out loss of co-REST in specific enhancers as the origin of the de-repression of gene expression and established that loss of LSD1 increases recruitment of the acetyltransferase p300/CBP to chromatin, possibly through competitive binding leading to increased transcription [25]. These findings were confirmed by an independent study, which also demonstrated that LSD1 acts as a scaffold to promote deubiquitylation of DNMT1 and UHRF1 independently of its catalytic activity, also promoting DNA methylation and transcriptional repression in this manner [26]. Another study in zebrafish confirmed that global gene repression regulation by LSD1 is independent of its catalytic activity but involves a scaffolding function mediated by the SNAG-binding domain of LSD1 [27]. Moreover, LSD1 was shown to interact with the Snail/Slug transcription factors through binding of its C-terminal amine oxidase-like (AOL) domain to the SNAG domain of the EMT promoting transcription factors [28]. The N-terminus of Snail binds the catalytic cleft of LSD1, preventing catalysis in an example of molecular mimicry to the H3 tail [29]. While LSD1/Snail interaction is necessary for co-REST recruitment to E-cadherin-regulatory elements, the requirement of the enzymatic activity of LSD1 is debatable since other repressive chromatin marks like histone deacetylation seem to be sufficient to repress expression of most LSD1-regulated genes [25]. Therefore, it is plausible that the increased expression of Snail and Slug in SSc fibroblasts contributes to a demethylase-independent pro-fibrotic function of LSD1.

Finally, our data demonstrate a role for LSD1 activity in maintenance of the pro-fibrotic phenotype of HOTAIR-expressing fibroblasts, which share some characteristics of SSc fibroblasts. However, we observed a lack of cooperation between LSD1 with the PRC2 in established SSc myofibroblasts. This suggests that while PRC2 is necessary for the stable maintenance of SSc myofibroblasts, LSD1/coREST plays a role in myofibroblast differentiation when triggered by diverse fibrotic stimuli.

One intriguing observation was the ability of LSD1 to regulate SMAD2/3 expression and activation in response to TGF-β. Inhibition of LSD1 reduced total SMAD3 levels in healthy and SSc dermal fibroblasts stimulated with TGF-β. Therefore, it is likely that the reduction in pSMAD3 by GSK-LSD1 in TGF-β-stimulated fibroblasts is due to reduced total SMAD3 levels. Interestingly, LSD1 inhibition does not reduce SMAD3 levels in unstimulated SSc fibroblasts, although it reduces pSMAD3. Also of interest, we observed no changes in pSMAD3 levels between scramble control and HOTAIR-expressing fibroblasts, suggesting that the ability of LSD1 to regulate the activation of SMAD3 in response to TGF-β stimulation is independent of HOTAIR. A potential explanation for the role of LSD1 on SMAD3 activation is that physical interaction of pSMAD3 and LSD1, as reported during EMT [30], protects pSMAD3 from dephosphorylation and/or degradation. LSD1 has previously been shown to stabilise oestrogen-related receptor (ERRα) by protecting them from proteasomal degradation [31], so a similar consequence on pSMAD3 is not unlikely. LSD1 could also modulate SMAD2/3 activity through regulating expression of genes involved in SMAD2/3 activation. For example, LSD1 regulates SEPT6 expression through its histone demethylase function, and this, in turn, activates SMAD2/3 [32].

Epithelial cells that undergo EMT are believed to be a major source for SSc myofibroblasts in the skin [33]. This is interesting as LSD1 regulates EMT in a range of cancers and in injury-induced tissue fibrosis. The previously mentioned scaffolding function of LSD1 binding the SNAG domain of Snail and the demethylase-independent role of LSD1 in SSc myofibroblasts may further strengthen the links between EMT in SSc.

Previously, we have shown that HOTAIR cooperates with the PRC2 complex to suppress the expression of miRNA-34a and activate NOTCH1 expression and signalling [8]. Here, we show that LSD1 activity can also enhance Notch signalling, as determined by the Notch target *HES1*; however, it does not affect NOTCH1 transcription. Interestingly, inhibition or silencing of LSD1 in oesophageal squamous cell carcinoma cell lines led to the downregulation of *NOTCH1*, *NOTCH3*, and *HES1*, and in small-cell lung cancer, it led to the downregulation of the Notch ligand *DLL4* [34,35], suggesting that LSD1 could modulate Notch signalling by mechanisms independent of HOTAIR.

The results of this study indicate that LSD1 contributes to pro-fibrotic gene expression in SSc by mechanisms mostly independent of HOTAIR.

## Data Availability

All data generated or analysed during this study are included in the published article.

## Abbreviations

SSc: systemic sclerosis; LSD1: lysine-specific histone demethylase 1; TGF-β: transforming growth factor beta; α-SMA: alpha smooth muscle actin; PRC2: polycomb repressor complex; EMT: epithelial-to-mesenchymal transition.
